# Curriculum Leadership of Rural Teachers: Status Quo, Influencing Factors and Improvement Mechanism-Based on a Large-Scale Survey of Rural Teachers in China

**DOI:** 10.3389/fpsyg.2022.813782

**Published:** 2022-03-11

**Authors:** Xinyu Wang, Junyuan Chen, Wei Yue, Yishi Zhang, Fenghua Xu

**Affiliations:** ^1^School of Education, Central China Normal University, Wuhan, China; ^2^School of Management, Wuhan University of Technology, Wuhan, China

**Keywords:** rural teachers, teacher curriculum leadership, teacher curriculum leadership views, teacher curriculum leadership practices, teacher curriculum leadership identity, teacher professional development

## Abstract

Revealing the general status quo of teacher curriculum leadership has great theoretical, policy, and practical significance. However, large-scale empirical investigations in this area are rare, and there is even less attention to the current situation of rural teacher curriculum leadership. Based on the survey of 2,966 rural teachers in 20 provinces of China, this paper presented the status quo of rural teacher curriculum leadership and examined influencing factors through multiple linear regression analysis. It was found that curriculum leadership of rural teachers was at a low level with backward leadership views, lack of practical ability, and low sense of identity. Regression analysis demonstrated that individual field factors had a significant impact on teachers’ curriculum leadership. Specifically, the higher the teachers’ leadership willingness, trust in others, and self-efficacy, the higher the curriculum leadership. The school field was also an important influential aspect. In particular, the formation of a common vision and teacher community by the school and the appropriate empowerment of the principal had a significant positive impact on the curriculum leadership of rural teachers. Based on these key findings, several improvement suggestions are put forward at the end, which can be used as references for other countries to develop improvement plans on rural teacher curriculum leadership.

## Introduction

Since the second half of the 20th century, teacher leadership has gradually developed into international discourse. Researchers in many countries have carried out a large number of empirical studies and model constructions on teacher leadership in combination with local and other countries’ realities. These studies covered a wide range of topics, among which the practical dimensions of teacher leadership (York-Barr and Duke, 2004; Teacher Leadership Exploratory Consortium, 2011)and its relationship with student development (Cheung et al., 2018; Shen et al., 2020) have received the most concentrated attention. A recent meta-analysis (Shen et al., 2020) has shown that among the seven areas of teacher leadership, curriculum, teaching, and evaluation improvement by teachers is most closely related to students’ academic achievements. It can be seen that in the context of teacher leadership, further focusing on the possible actions of teachers in the curriculum field is of great significance in theory, policy, and practice.

Perhaps it is for this consideration that in recent years, many researchers have begun to put the research horizon of teacher leadership in the field of curriculum, that is, teacher curriculum leadership. Combining the definition of teacher leadership and curriculum leadership by related scholars, this study believes that teacher curriculum leadership is the capacity of teachers to cooperate with stakeholders in the curriculum field to better promote student development. Generally speaking, focusing on the curriculum leadership of teachers, scholars have made explorations mainly from four aspects:

The first aspect was the empirical verification of the necessity of teacher curriculum leadership. A longitudinal study of the ninth-grade Integrated Science Curriculum (ISP) developed and implemented by teachers of the science department of a public high school in a metropolitan school district in the southwestern United States for more than 25 years showed that ISP is the product of teacher curriculum leadership. It reflected how teachers with curriculum leadership respond to the needs of differentiated students without sacrificing the essential characteristics of the curriculum (Larkin et al., 2009). Some researchers have also carried out a case study of how multi-grade teachers overcame teaching difficulties of a rural primary school in South Africa and found that teachers were facing a lack of appropriate training and workshops, insufficient support from stakeholders, and lack of school resources, challenges as such made it more difficult to teaching and learning. However, these teachers adopted teaching leadership to adapt to the teaching environment, so that they could overcome the challenges of teaching in an environment where resources were scarce and neglected, thus confirming the reality that teaching leadership was a key driving factor for multi-grade classroom teaching (Ramrathan and Ngubane, 2013).

The second aspect was the case analysis of teachers’ curriculum leadership path and level (Ritchie et al., 2006; Lai and Cheung, 2015; Meirink et al., 2020). A study focused on the level of informal curriculum leadership implemented by 12 novice teachers working in middle schools in different regions of Netherlands. These novice teachers showed three levels of curriculum leadership: witness, participation, and ownership, which also proved the possibility of novice teachers to assume curriculum leadership. However, novice teachers needed to develop leadership knowledge and skills for this purpose (Meirink et al., 2020). Another study revealed the leadership practices of teachers in implementing curriculum reform. Based on interview data of teachers from 9 high schools in Hong Kong, China, this article has identified three paths of teacher curriculum leadership in several major areas such as participation in school-based curriculum planning, meeting the diverse needs of learners, teacher learning, and teacher influence, namely complying with external requirements, adapting current practices to accommodate reform requirements, and empowering school changes (Lai and Cheung, 2015).

The third aspect was the clarification of obstacles and contributions to teacher curriculum leadership (Jita and Matseliso, 2013; O’Gorman and Hard, 2013; Clohessy et al., 2020). A study examined the impediments and enablers of distributed leadership among teachers in Queensland, Australia. In the reflections of the 13 teachers participating in the survey, the researchers determined that resources, materials, values, and ideas are the structural elements that encourage or restrict them to enact distributed leadership. Among them, obstacles to teacher curriculum leadership were lack of respect from principals or encountering resistance from colleagues when introducing new programs or curricula to schools, facilitating factors were teachers’ interpersonal skills, creativity, sensitivity and advocacy (O’Gorman and Hard, 2013). Another study showed the leadership of physical education teachers in class swapping in two urban primary schools in Ireland. Using qualitative data generated by personal interviews, student focus groups, researchers’ observations, and teachers’ reflection, the study examined the 18-week class swapping experience of two generalist primary school teachers and found that teaching physical education to classes other than their own increased teachers’ ability and motivation to provide further curriculum leadership within their schools (Clohessy et al., 2020).

In addition, some studies have explored future teachers’ conceptions of curriculum leadership from the perspective of teacher education. A phenomenological study of 24 future teachers in Hong Kong, China identified three conceptual differences in curriculum leadership: curriculum leadership as individual leadership, curriculum leadership as hierarchy, and curriculum leadership as a network. The study pointed out that different conceptions revealed the “choice of focus” of future teachers, which might determine how they learn and implement curriculum leadership in school. To this end, the concept of curriculum leadership of future teachers needed to be considered when planning and organizing teacher education programs related to curriculum development and implementation (Wan and Leung, 2021).

Researches above have a concern about the actual situation of the use of teacher curriculum leadership and provided a precious starting point for follow-up research. However, although the existing studies focused on the diversity of the backgrounds of the research subjects, their analyses still focused on generalizing the commonalities of curriculum leadership among urban and rural teachers, blurring the differences between urban and rural areas (Ritchie et al., 2006; O’Gorman and Hard, 2013; Meirink et al., 2020). However, more studies have revealed the objective existence of differences between urban and rural areas, but there are disputes between the conclusions. A survey of 492 urban teachers and 423 rural teachers in 31 schools in three Chinese provinces revealed significant differences in learning-centered leadership and teacher learning between urban and rural schools, with urban schools having significantly higher levels of all variables. Rural teacher samples tend to be younger, lower in educational attainment, and less engaged in in-service learning than urban teachers. This difference explains, in part, the difference in academic achievement between urban and rural students (Hallinger and Liu, 2016). But rural teachers are not always at a disadvantage. A survey of 368 student teachers at a mid-sized Midwestern university showed that urban and rural school environments affect student teachers’ perceptions of efficacy. Although each student teacher’s sense of efficacy improved significantly at the end of the 16-week teaching experience. However, in comparison, student teachers in urban schools had significantly lower efficacy than student teachers in rural schools (Knoblauch and Chase, 2015). To some extent, this study breaks the stereotype of rural teachers and rural education, that is, rural areas are a problem field and rural areas are equivalent to backwardness (Theobald, 2005). In view of such different conclusions about urban and rural areas, some studies have noted the polarization phenomenon in rural studies, with romance at one pole and defect at the other pole. This single perspective hinders the deep cognition of complex rural situations (Burton et al., 2013; Heppner, 2017). Therefore, when studying rural teachers, it is especially important to put aside preconceived biases about rural areas and try to reveal the current situation of rural teachers as objectively as possible. This is of great significance for fully understanding rural teachers and for correctly perceiving the differences between rural and urban teachers.

This study aims to enhance rural teacher research by investigating the status quo of curriculum leadership and the influencing factors among primary and secondary school teachers in rural China. The number of primary and secondary school teachers in China ranks among the highest in the world, among which rural teachers are an important force. According to education statistics in 2019, the total number of full-time teachers in primary and secondary schools in China was 11,875,755, among which, the number of full-time teachers in rural areas was 2,447,692, accounting for 20.61% (Ministry of Education of the People’s Republic of China, 2020a,b,c). In recent years, the Chinese government has comprehensively promoted rural revitalization and has made “prioritizing the development of rural education and building a strong rural teacher team” (Central Committee of the Communist Party of China and the State Council, 2018) as an important part of implementing the rural revitalization strategy. In this context, China issued the special document *Circular on Reinforcing Rural Teacher Development in the New Era* (Ministry of Education of the People’s Republic of China et al., 2020) and *Targeted Training Plan for Outstanding Teachers in Underdeveloped Areas in Central and Western China* (Ministry of Education of the People’s Republic of China et al., 2021), which reflected a great emphasis on rural teachers’ quality. In this view, it is of great policy significance to investigate the current situation of rural primary and secondary school teachers’ curriculum leadership in China. This study focuses on the group of teachers in rural primary and secondary schools in China, and specifically explores the following two questions:

RQ1: What is the realistic level of curriculum leadership for teachers in rural primary and secondary schools in China?
RQ2: What factors have affected this status quo?

## Materials and Methods

This study used quantitative methods to analyze the general status quo of rural teachers’ curriculum leadership in China. Based on the literature review, a theoretical framework was established and a questionnaire was developed. And through a large-scale survey across the country, understanding of the current level of curriculum leadership and its influencing factors of Chinese rural teachers in the context of the new curriculum reform based on key competencies was deepened.

### Data Source and Participants

The data used in this study was derived from an empirical investigation by “Study on Curriculum Leadership of Primary and Secondary School Teachers from the Perspective of Key Competencies” Research Group from January 2020 to April 2021 in 20 provinces of China including Sichuan, Hubei, Guangxi, Shandong, Shanxi, Hunan, Beijing, Ningxia, Guangdong, Xinjiang, Zhejiang, Jiangsu, Guizhou, Chongqing, Hebei, Henan, Shanghai, Anhui, Inner Mongolia, and Qinghai. In this study, the stratified random sampling method was used to randomly select primary schools, junior middle schools, and ordinary high schools in 20 provinces according to the geographical location and economic development level of each region. A questionnaire survey was conducted among all teachers in the sampled schools. In terms of the survey method, considering the Epidemic situation, data collection was carried out with the help of an online electronic questionnaire (“Questionnaire Star”). The entire collection process followed the principles of voluntariness and anonymity. In the end, 23,915 questionnaires were returned, of which 19,521 were valid (response rate = 81.63%). According to the research needs, 2,966 rural teachers were selected from the total sample as the research participants. Table 1 presents the demographic characteristics of the selected rural teachers’ sample.

**TABLE 1:** Demographic characteristics of 2,966 rural teachers.

Characteristics	Number of People	Percentage (%)	Characteristics	Number of People	Percentage (%)
**Gender**			**Position**		
Female	1,826	61.6	With position (s)	2,107	71.0
Male	1,140	38.4	With no position	859	29.0
**Teaching age**			**School ranking**		
0–5 years	883	29.8	Medium and below medium	1,166	39.3
6–10 years	283	9.5	Above medium	1,629	54.9
11–15 years	194	6.5	The best	171	5.8
16–20 years	234	7.9	Job title		
21 years and above	1,372	46.3	Unrated	426	14.4
**School location**			Third-level teacher	120	4.0
Ethnic regions	1,058	35.7	Secondary teacher	823	27.7
Non-ethnic regions	1,908	64.3	First-level teacher	1,135	38.3
**School nature**			Advanced teacher	461	15.5
Public school	2,928	98.7	Senior teacher	1	0.1
Private school	38	1.3	Teacher education background		
**Teaching period**			Yes	2,504	84.4
Primary school	1,936	65.3	No	462	15.6
Middle school	987	33.3	Highest degree		
High school	43	1.4	Below college degree	39	1.3
**Teaching subject**			College degree	876	29.5
Single discipline	1,777	59.9	Bachelor degree	2,010	67.8
Multiple disciplines	1,189	40.1	Above bachelor degree	41	1.4

### Research Instruments

#### Instruments and Procedures

The questionnaire was divided into three parts (see Supplementary Appendix A). PART I was the demographic information of rural teachers, corresponding to item 1–12 of the questionnaire. This part was set to show the basic information of teachers, for example, teachers’ gender, teaching age, professional background, highest degree, and so on. Most of these variables have been shown to have an impact on teachers’ curriculum leadership (e.g., Karachiwalla and Park, 2017), but were not the focuses in this study, so they were used as control variables. PART II was a measure of the dependent variable—the rural teachers’ curriculum leadership. According to the teacher leadership model constructed by Sinha and Hanuscin (2017), it was divided into three sub-dimensions: teacher curriculum leadership views, teacher curriculum leadership practices, and teacher curriculum leadership identity (Xu and Chen, 2021). These were measured by item 14 (1–29) of the questionnaire. PART III was to measure the independent variables, namely the influencing factors of teacher curriculum leadership. This part was based on Lewin’s field dynamic theory (Lewin, 1997; Burnes and Cooke, 2013), and was measured from two fields: the individual field and the school field, corresponding to item 13 and item 14 (30–42) of the questionnaire. Except that PART III contained a single choice question, both PART II and PART III adopted the commonly used Likert five-point scales. Since there was no ready-made measurement tool for reference, this study developed a questionnaire based on relevant literature (Silva et al., 2000; Komives et al., 2005; Chen Cravens, 2014; National Survey Research Center at Renmin University of China, 2014; Hunzicker, 2017; Sinha and Hanuscin, 2017; Teaching and Research Office of Shanghai Municipal Education Commission, 2019).

To ensure the content validity of the questionnaire, five experts in the field of education and teaching were invited to carry out the content evaluation. Four experts are university teachers, they come from different disciplines and have their expertise in teacher education, rural education, curriculum and pedagogy, and statistics, respectively. Another expert is a nationally renowned high school teacher as well as a teaching-research officer of the Municipal Academy of Education Sciences, whose research expertise is high school mathematics (see Table 2). All of these experts had rich teaching and research experience and had a profound understanding of primary and secondary school education practices and teachers. These five experts made a comprehensive evaluation on the correspondence between the questionnaire and the indicators, the scientificity of the questionnaire structure, the rationality of the questionnaire items, and the appropriateness of their expressions. It was found that the questionnaire could better reflect the research content. The researchers revised the expressions of some items based on expert suggestions. For example, since the participants of the survey were teachers across the country, this inevitably involved the issue of urban-rural differences. Therefore, under the advice of experts, our items not only considered the general needs of the national education development but also tried our best to take into account the applicability of the questionnaire to both rural and urban teachers. Subsequently, researchers in this study conducted a trial survey on the revised questionnaire in four schools, refined the description of items based on teachers’ feedback, and added two new items. The final questionnaire contains 55 questions, 13 of which are multiple-choice and fill-in-the-blank questions, 42 items are in scale form, all of which are evaluated with Likert five-point scale ranging from “strongly disagree” (1) to “strongly agree” (5). Cronbach’s α was used to measure the internal consistency of the items in the scale. The reliability analysis results show that the reliability coefficients of the total scale and subscale are between 0.826 and 0.974, indicating good internal consistency (see Table 3).

**TABLE 2:** The qualifications of the experts.

Expert number	Profession	Expertise background
1	University Teacher	Teacher education
2	University Teacher	Rural education
3	University Teacher	Curriculum and pedagogy
4	University Teacher	Statistics
5	High school teacher and teaching-research officer	High school mathematics

**TABLE 3:** The reliability test of teacher curriculum leadership scale.

Measurement dimension	Internal consistency reliability test (Cronbach’s α)	Number of items on the scale (N)
Teacher curriculum leadership views	0.949	5
Teacher curriculum leadership practices	0.946	20
Teacher curriculum leadership identity	0.826	4
Teacher curriculum leadership	0.964	29
Individual field factors	0.881	5
School field factors	0.953	8
Influencing factors of teacher curriculum leadership	0.952	13
Total scale	0.974	42

#### Dependent Variable

The dependent variable in this study is teacher curriculum leadership. Sinha and Hanuscin (2017) creatively proposed the three-dimension model of teacher leadership, pointing out that teacher leadership should include three aspects: teacher leadership views, teacher leadership practices, and teacher leadership identity. Referring to this model of teacher leadership and combining with the analysis of relevant literature, the questions in this part of the questionnaire were compiled from three dimensions, namely teacher curriculum leadership views (Fan, 2013), teacher curriculum leadership practices (York-Barr and Duke, 2004; Chang et al., 2011; Teacher Leadership Exploratory Consortium, 2011; Chen Cravens, 2014; Liu et al., 2016; Teaching and Research Office of Shanghai Municipal Education Commission, 2019), and teacher curriculum leadership identity (Silva et al., 2000; Komives et al., 2005). When analyzing the influencing factors of teacher curriculum leadership, this study not only took the overall teacher curriculum leadership as the dependent variable but also explored the influence of the individual field factors and the school field factors on teacher curriculum leadership views, practices, and identity. Since China has initiated a new round of basic education curriculum reform driven by key competencies in 2014 and updated the ordinary high school curriculum plan and curriculum standards accordingly, this study used key competencies as a macro background for guiding the development of the questionnaire and penetrated it into the expressions of specific items. It can be seen from Table 3 that all of the scales have good internal consistency.

#### Independent Variables

According to Lewin’s field dynamic theory (Lewin, 1997; Burnes and Cooke, 2013; Chen et al., 2021), possible factors affecting teacher curriculum leadership should be explored from two aspects: the teacher individual field and the school field (teachers’ daily life space). Combined with relevant literature, we refined the indicators and items contained in each aspect.

Individual field factors: Through a review and analysis of relevant literature, the researchers selected 6 variables in this field: professional level (York-Barr and Duke, 2004), leadership willingness (Harris and Muijs, 2005), trust quality (Brosky, 2009), self-efficacy (Durias, 2010), self-planning and management ability (O’Gorman and Hard, 2013), interpersonal skills (Muijs and Harris, 2003). The professional level here was a dummy variable, represented by the highest level of education and teaching awards that teachers have received so far, with “not yet awarded” as the reference group. Based on the remaining five variables, a subscale consisting of five items was constructed. The internal consistency reliability coefficient for the scale was 0.881.

School field factors: In the systematic review of Childs-Bowen et al. (2000), Brooks et al. (2004), York-Barr and Duke (2004), Muijs and Harris (2006), Mangin (2007) and other documents, combined with the results of expert consultation, the researchers selected 4 dimensions including school culture, teacher community, school organizational structure and principal, and developed a subscale with 8 items. Among them, the school culture dimension mainly observes whether the school has formed a cultural atmosphere of common development vision and trust (Brooks et al., 2004; Muijs and Harris, 2006; Daly, 2008); the teacher community dimension aims to examine the degree of teacher collaboration and sharing in the process of rural teachers’ curriculum leadership (Margolis and Doring, 2012); the dimension of school organizational structure mainly measures whether the school has formed a democratic mechanism that allows teachers to participate in curriculum decision-making (Pellicer and Anderson, 1995; Frost and Harris, 2003). The principal factor is relatively complex. Based on existing research results, this dimension can be divided into principal’s support for teacher professional development (Beachum and Dentith, 2004), the communication between principals and teachers (Gordin, 2010), and principal’s empowerment of teachers (Starr, 2019; Celik and Konan, 2021) and principal’s rewards for teachers (Borchers, 2009). The internal consistency reliability coefficient for the scale was 0.953.

### Data Analysis

With the help of SPSS V. 22.0, the collected data of 2,966 rural teachers in China was analyzed statistically. Firstly, descriptive statistics were used to describe the mean and standard deviation of rural teachers’ curriculum leadership in the overall and sub-dimensions. Secondly, the independent sample *T*-test was used to determine the relative level of rural teachers by comparing the difference in curriculum leadership development level between urban and rural teachers in China. Finally, the multiple linear regression analysis was adopted to explain the factors affecting the development of rural teachers’ curriculum leadership. To be concrete, by controlling variables such as gender, teaching age, educational background, professional titles, a linear regression equation between rural teachers’ individual field factors as well as school field factors and teacher curriculum leadership was set up. According to the significance and standardized regression coefficient β, factors playing a key role in the development of rural teacher curriculum leadership were made clear. The equation expression of the econometric regression model is:


Y=jα+βX1+1…+βX14+14βX15+15…+βX25+25ε

In this model, Y_j_ represents the dependent variable of rural teachers’ curriculum leadership. X_1_ to X_14_ represent in turn the independent variables of professional level, willingness to lead, trust, self-efficacy, self-planning and management ability, interpersonal skills, a common vision of the school, school cultural atmosphere, teacher community, school organization structure, principal support for teachers, principal-teacher communication, principal empowerment of teachers, principal reward to teachers. X_15_ to X_25_ represent the control variables, including gender, teaching age, professional background, highest degree, position, professional title, ethnic location, school nature, school ranking, teaching subject, teaching period, and ε is the error term. When exploring factors affecting the views, practices, and identity of rural teachers’ curriculum leadership, the equation was the same.

## Results

### Analysis on the Overall Level of Rural Teachers’ Curriculum Leadership

This study conducted an independent sample *T*-test on the curriculum leadership level of rural teachers and urban teachers, finding that in addition to post identity, rural teachers in curriculum leadership overall level, curriculum leadership views, practices, and identity as well as sub-indicators of the three main dimensions showed a significant difference (*p* < 0.001). From the average point of view, compared with urban teachers, rural teachers’ curriculum leadership level was also significantly lower in all dimensions (see Table 4), which revealed the urgent need to improve rural teachers’ curriculum leadership. From the perspective of rural teachers’ own curriculum leadership level, scores on curriculum leadership views were higher than that of curriculum leadership practices and identity, indicating that rural teachers had a certain understanding of curriculum leadership, but their practical ability was relatively insufficient, let alone internalizing it as an identity. Specifically:

**TABLE 4:** The overall level of rural teachers’ curriculum leadership.

Variables	Full sample		Rural teachers		Urban teachers		*T* test
	Mean	SD	Mean	SD	Mean	SD	
The overall level of teacher’s curriculum leadership	3.76	0.577	3.67	0.553	3.78	0.579	9.246***
Teacher curriculum leadership views	3.83	0.636	3.73	0.618	3.85	0.637	8.846***
Factual idea	3.85	0.636	3.75	0.624	3.87	0.636	10.057***
Methodological concept	3.77	0.718	3.69	0.683	3.78	0.723	5.954***
Value concept	3.82	0.707	3.73	0.679	3.83	0.711	6.941***
Teacher curriculum leadership practices	3.76	0.594	3.66	0.571	3.78	0.597	10.319***
Curriculum thought	3.78	0.634	3.69	0.604	3.80	0.637	8.981***
Cultural modernity	3.68	0.715	3.57	0.689	3.70	0.718	9.007***
Policy understanding	3.82	0.658	3.73	0.632	3.84	0.661	8.002***
Vision consistency	3.81	0.717	3.71	0.704	3.83	0.718	8.188***
Curriculum design	3.89	0.645	3.79	0.629	3.90	0.646	8.767***
Overall planning	3.90	0.687	3.80	0.673	3.90	0.691	7.602***
Resources consciousness	3.90	0.688	3.82	0.682	3.92	0.687	7.233***
Collective lesson preparation	3.87	0.703	3.76	0.694	3.89	0.703	9.412***
Curriculum implementation	3.95	0.583	3.85	0.566	3.97	0.584	10.277***
Student-oriented	3.96	0.618	3.86	0.607	3.98	0.618	9.561***
Professional support	3.89	0.646	3.78	0.632	3.91	0.647	9.878***
Dynamic generation	4.08	0.634	3.99	0.627	4.10	0.634	9.087***
Curriculum evaluation	3.91	0.609	3.81	0.591	3.93	0.610	10.489***
Clear guidance	3.88	0.663	3.79	0.647	3.90	0.665	8.963***
Timely monitoring	3.89	0.645	3.78	0.628	3.91	0.646	9.901***
Effective improvement	4.00	0.636	3.88	0.629	4.02	0.635	10.508***
Curriculum development	3.24	1.166	3.12	1.429	3.26	1.685	6.048***
Response to needs	3.36	1.191	3.23	1.177	3.38	1.192	6.230***
Appropriate resources	3.32	1.217	2.30	1.196	3.34	1.220	6.117***
Due procedure	3.15	1.232	3.03	1.205	3.17	1.236	5.548***
Teacher curriculum leadership identity	3.71	0.645	3.63	0.626	3.72	0.648	6.735***
Group identity	3.85	0.702	3.75	0.686	3.86	0.703	8.132***
Self-identity	3.66	0.780	3.58	0.760	3.67	0.783	5.627***
Post identity	3.47	0.985	3.45	0.910	3.47	0.998	1.370
Responsibility identity	3.86	0.727	3.76	0.714	3.88	0.727	8.530***

***p < 0.001.

#### Rural Teachers’ Curriculum Leadership Views Were Backward

From the perspective of teacher curriculum leadership views, compared with urban teachers, rural teachers lagged in the three dimensions of factual views, methodological views, and value views (*P* < 0.001). This revealed that rural teachers fundamentally lacked the understanding of the connotation and value of curriculum leadership, and they didn’t have a good understanding of what curriculum leadership was, what significance curriculum leadership had for student development, and how to exert curriculum leadership. It indicated that rural teachers’ understanding of teacher curriculum leadership needed to be improved.

#### Rural Teachers Lacked the Practical Ability of Curriculum Leadership

From the perspective of teacher curriculum leadership practices, there were significant differences between rural teachers and urban teachers in curriculum thought, curriculum design, curriculum implementation, curriculum evaluation, and curriculum development, indicating that rural teachers had a low level of curriculum leadership practices and lacked practical quality in implementing curriculum leadership.

Through in-depth analysis of the third-level indicators, this study found that in terms of curriculum thought, rural teachers and urban teachers had a clear gap in the 3 indicators of cultural modernity, policy understanding, and vision consistency, reflecting the rural teachers’ mastery of the frontier education ideas and national policies was not timely and failed to exert their charm to lead parents and colleagues to build curriculum vision. In terms of curriculum design, there were significant differences between the two types of teachers in the overall planning, resource consciousness, and collective lesson preparation. It could be seen from the data that the performance of rural teachers in curriculum design was even worse, indicating that compared with urban teachers, rural teachers often lacked an overall understanding of students and the curriculum they taught, and were unable to make curriculum planning accordingly. At the same time, rural teachers didn’t make full use of the existing material and human resources, failing to fully integrate school, community, Internet, and other curriculum resources into the curriculum service system. They also neglected to strengthen communication and sharing with colleagues or improve curriculum planning relying on the power of the professional community. In terms of curriculum implementation, rural teachers’ performance in the 3 indicators of student-oriented, professional support, and dynamic generation was relatively weak, indicating that rural teachers tended to ignore the interaction with students in class and didn’t pay enough attention to the needs of students. Moreover, teachers still tended to use traditional ways in the curriculum rather than modern information technology to guide students to achieve autonomous development, and the teaching lacked flexibility. In terms of curriculum evaluation, the two types of teachers also showed significant differences in 3 indicators, namely clear guidance, timely monitoring, and effective improvement, which demonstrated that rural teachers lacked clear awareness of curriculum evaluation orientation and were not good at evaluating, reflecting and adjusting according to the actual progress of curriculum. In the aspect of curriculum development, rural teachers lacked enthusiasm and professionalism in developing school-based curriculum.

#### Rural Teachers’ Sense of Identity in Curriculum Leadership Was Not Strong

From the perspective of teacher leadership identity, in addition to post identity, there were significant differences in group identity, self-identity, and responsibility identity between rural teachers and urban teachers. According to the mean value, rural teachers didn’t agree with the idea that “teachers as a professional group can and should participate in curriculum leading, management and decision-making.” Besides, the rural teacher had lower self-identity and lacked confidence in participating in curriculum leadership. However, there was no significant difference between the two types of teachers in the dimension of post identity. It could be seen that they held the same opinion on “only with administrative positions can teachers enact curriculum leading, management, and decision making,” that was, both groups believed that the post was not a necessary condition for teachers to enact curriculum leadership. In other words, whether teachers had administrative positions, they were “curriculum leaders” if they could actively participate in the construction of the curriculum community.

### Analysis of the Influencing Factors of Rural Teachers’ Curriculum Leadership

As mentioned above, rural teachers were in a weak position in all dimensions of curriculum leadership. Based on this, it was necessary to investigate the causes and clarify the main factors affecting the development of curriculum leadership of rural teachers to find a breakthrough for effective improvement. Therefore, this study adopted a multiple linear regression model (see Table 5) to analyze the influence of teachers’ individual field factors and school field factors on the overall level of rural teachers’ curriculum leadership (Model 1) and the three key dimensions (Model 2, 3, and 4) by controlling variables successively.

**TABLE 5:** Analysis results of influencing factors of rural teachers’ curriculum leadership.

	Model 1	Model 2	Model 3	Model 4
	
	Teacher curriculum leadership	Teacher curriculum leadership views	Teacher curriculum leadership practices	Teacher curriculum leadership identity
**Individual field factors**				
Professional level-School level awards	0.007 (0.023)	0.018 (0.028)	0.004 (0.027)	0.002 (0.026)
Professional level-County (district) level awards	−0.009 (0.021)	0.007 (0.025)	−0.010 (0.024)	−0.023 (0.024)
Professional level-Municipal awards	0.019 (0.023)	−0.005 (0.028)	0.029 (0.027)	−0.004 (0.027)
Professional level-Provincial awards	0.014 (0.030)	−0.001 (0.037)	0.023 (0.036)	−0.015 (0.035)
Professional level-National awards	0.017 (0.032)	0.016 (0.039)	0.021 (0.038)	−0.009 (0.037)
Leadership willingness	0.290*** (0.009)	0.221*** (0.011)	0.253*** (0.011)	0.433*** (0.011)
Trust quality	0.143*** (0.013)	0.190*** (0.016)	0.115*** (0.015)	0.158*** (0.015)
Teacher self-efficacy	0.081*** (0.014)	0.108*** (0.017)	0.065*** (0.017)	0.091*** (0.016)
Teacher self-planning and management ability	0.055*** (0.014)	0.089*** (0.017)	0.046* (0.016)	0.031 (0.016)
Interpersonal skills	0.177 (0.014)	0.111*** (0.017)	0.192*** (0.016)	0.119*** (0.016)
**School filed factors**				
School common vision	0.140*** (0.016)	0.157*** (0.019)	0.134*** (0.019)	0.091*** (0.018)
School culture	0.020 (0.016)	0.052* (0.020)	0.017 (0.019)	−0.016 (0.019)
Teacher community	0.053** (0.015)	0.023 (0.018)	0.065** (0.017)	0.014 (0.017)
School organizational structure	−0.013 (0.015)	−0.010 (0.018)	−0.026 (0.018)	0.048* (0.017)
Principal support	0.014 (0.017)	0.022 (0.021)	0.002 (0.020)	0.053* (0.020)
Principal-teacher communication	0.015 (0.016)	−0.005 (0.020)	0.041 (0.019)	−0.085*** (0.018)
Principal empowerment	0.057** (0.014)	0.031 (0.017)	0.059** (0.016)	0.058** (0.016)
Principal reward	0.021 (0.012)	0.018 (0.015)	0.016 (0.014)	0.038* (0.014)
**Control variables**				
Gender-Male	−0.011 (0.013)	0.014 (0.016)	−0.017 (0.015)	−0.012 (0.015)
Teacher education background-Yes	0.022* (0.016)	0.025* (0.020)	0.023 (0.019)	0.008 (0.019)
Teaching age-6–10 years	−0.002 (0.022)	0.002 (0.027)	−0.005 (0.026)	0.006 (0.026)
Teaching age-11–15 years	0.001 (0.027)	−0.001 (0.033)	0.000 (0.032)	0.004 (0.031)
Teaching age-16–20 years	0.022 (0.028)	0.019 (0.034)	0.024 (0.033)	0.007 (0.033)
Teaching age-21 years and above	0.075*** (0.025)	0.071** (0.031)	0.080** (0.029)	0.030 (0.029)
Teaching period-Middle school	−0.014 (0.013)	−0.008 (0.016)	−0.015 (0.015)	−0.014 (0.015)
Teaching period-High school	0.010 (0.048)	0.303** (0.058)	0.003 (0.056)	0.010 (0.055)
Teaching subject-Multidisciplinary	−0.007 (0.012)	−0.017 (0.015)	−0.004 (0.014)	−0.006 (0.014)
Highest degree-Below college degree	−0.013 (0.050)	−0.031** (0.061)	−0.009 (0.059)	0.000 (0.057)
Highest degree-College degree	−0.029* (0.014)	−0.015 (0.017)	−0.033* (0.016)	−0.017 (0.016)
Highest degree-Above bachelor degree	0.026* (0.048)	0.024* (0.058)	0.026* (0.056)	0.016 (0.055)
Title-Third-level teacher	−0.010 (0.032)	−0.006 (0.039)	−0.016 (0.038)	0.017 (0.037)
Title-Secondary-level teacher	−0.002 (0.022)	−0.020 (0.027)	−0.002 (0.026)	0.019 (0.026)
Title-First-level teacher	−0.056* (0.029)	−0.071* (0.036)	−0.057 (0.034)	−0.013 (0.034)
Title-Advanced teacher	−0.050* (0.033)	−0.074** (0.040)	−0.046 (0.039)	−0.019 (0.038)
Title-Senior teacher	0.006 (0.303)	0.000 (0.371)	0.007 (0.358)	0.005 (0.350)
Post-With administrative position (s)	−0.009 (0.013)	−0.007 (0.016)	−0.005 (0.015)	−0.026* (0.015)
School location-Ethnic	0.014 (0.012)	−0.014 (0.014)	0.019 (0.014)	0.021* (0.013)
School nature—Public	−0.011 (0.049)	−0.008 (0.060)	−0.013 (0.058)	0.000 (0.057)
School ranking—Medium and below	−0.008 (0.025)	0.052* (0.031)	−0.018 (0.030)	−0.033 (0.029)
School ranking-Above medium	−0.002 (0.024)	0.031 (0.030)	0.000 (0.029)	−0.051* (0.028)
Sample size	2,966			
Adjusted *R*^2^	0.707	0.649	0.616	0.696

*p < 0.05, **p < 0.01, and ***p < 0.001. The number in the bracket denotes the standard error.

#### Specific Factors Affecting the Overall Level of Rural Teachers’ Curriculum Leadership

The regression analysis results of Model 1 show that teachers’ individual field factors play a core role in affecting rural teachers’ curriculum leadership, that is, rural teachers’ leadership willingness, trust quality, self-efficacy, and interpersonal skills have a significant positive impact on their curriculum leadership (*p* < 0.001). In other words, teachers as the main subject of curriculum leadership would improve their curriculum leadership level and quality of curriculum construction to a great extent if they actively participated in curriculum leadership and established a good cooperative relationship with their colleagues. School field factors were the external elements that affected rural teachers’ curriculum leadership. Within 8 sub-dimensions of school field factors, common vision, teacher community, and principal empowerment were significantly correlated with curriculum leadership of rural teachers. This showed that, on the one hand, in addition to teachers’ own efforts, improving rural teachers’ curriculum leadership needed emotional support from the school’s common vision and teacher community. On the other hand, rural teachers’ curriculum leadership needed appropriate independent space. The more autonomy principals gave teachers in curriculum, the higher the level of teacher curriculum leadership.

#### The Realistic Mechanism of Impacting the View Level of Rural Teachers’ Curriculum Leadership

Consistent with the research results of Model 1, the results of Model 2 show that teachers’ individual field factors and school field factors have a significant impact on rural teachers’ views of curriculum leadership. Teachers’ individual field factors had a highly positive correlation with the curriculum leadership concept of rural teachers at the significance level of 0.1%. Further comparing the standardized coefficients of each sub-dimension, it was found that the willingness of leadership (β = 0.221) had the most prominent influence on rural teachers’ curriculum leadership cognition, indicating that when a teacher had a strong willingness to lead, it would internally stimulate the formation of the concept of teacher curriculum leadership. Among the school field factors, the teacher’s views of curriculum leadership were mainly affected by the school’s common vision and cultural atmosphere. If the school formed a consistent development goal and created a harmonious, progressive, united, and cooperative cultural atmosphere, it could effectively promote the transformation of rural teachers’ concept of curriculum leadership.

#### Internal and External Regulations That Influence the Practices of Rural Teachers’ Curriculum Leadership

Model 3 presents the influence of the individual and the school field factors on rural teachers’ curriculum leadership practices. In terms of teachers’ individual field factors leadership willingness, trust quality, and interpersonal skills were all significant at the level of 0.1%. Compared with Model 2, teachers’ leadership willingness and interpersonal skills have a greater impact on their curriculum leadership practices, indicating that although leadership willingness can promote the generation of rural teachers’ curriculum leadership concepts, it is more important to further guide teachers in curriculum design, implementation, and evaluation. In terms of school field factors, in addition to being affected by the school common vision, teachers’ curriculum leadership practices also presented a significant positive correlation with teacher community and principal empowerment (*p* < 0.1), demonstrating that promoting mutual communication and relevant cooperation with colleagues had an active effect on the improvement of rural teachers’ curriculum leadership practices. Principal empowerment also had a significant influence on curriculum leadership practices of rural teachers (β = 0.059), inferior only to school common vision and teacher community.

#### The Main Factors Affecting the Identification of Rural Teachers’ Curriculum Leadership

Model 4 reflects the influencing factors of rural teachers’ curriculum leadership identity. In terms of teachers’ individual field factors, the willingness of leadership had the greatest influence (β = 0.433), followed by trust quality (β = 0.158), and teachers’ interpersonal skills were the least (β = 0.119). Based on the above analysis, it could be concluded that leadership willingness was the root and driving force for the improvement of rural teachers’ curriculum leadership, which could further strengthen teachers’ internal identity based on promoting the development of teachers’ views and practices of curriculum leadership. In terms of school field factors, school common vision, school organizational structure, principal support, and principal communication had varying degrees of influence on teachers’ curriculum leadership identity. It was worth investigating that there was a significant negative correlation between principal-teacher communication and rural teachers’ curriculum leadership identity. The possible reason might be that part of principals managed curriculum relying on administrative power and work experience, which leads to certain resistance of teachers, thus reducing rural teachers’ sense of identity to the group and to themselves.

## Discussion

Due to the lack of large-scale empirical studies, we knew little about the general status quo of rural teacher curriculum leadership. To make up for this deficiency, this study focused on exploring the realistic level of rural teacher curriculum leadership and its influencing factors and made responses by surveying 2,966 rural teachers in primary and secondary schools in China. This section will combine the key research findings and carry out a dialog with related research, to deepen the relevant understanding.

### Rural Teachers’ Curriculum Leadership Was Weak and Needed to Be Improved Comprehensively

This study found that the curriculum leadership of rural teachers was generally at a low level, and there were obvious problems such as backward leadership views, lack of practical ability, and low degree of identity, which had been supported by previous studies. Song (2010) studied the current situation of curriculum leadership of rural teachers in Western China through questionnaire surveys and interviews and found that teachers in this region had low curriculum leadership awareness, lacked professional training, and owned low curriculum leadership ability. The possible explanation for the problems above is that, on the one hand, since the basic education curriculum reform in 2001, teachers were required to put students at the center of learning, promote students’ learning through praise and encouragement, and try new teaching methods, to change the classroom experience (Yiu and Adams, 2013). However, rural teachers are still bound by traditional teaching concepts, and continue to play the role of “loyal curriculum implementers.” In the curriculum leadership, rural teachers are completely unaware that they are independent and complete individuals as “teachers,” and at the same time are multiple roles of the developers of curriculum resources, the helpers of student learning, the collaborators of peer teachers, and the promoters of school development. They are immersed in their “comfort zone,” which affects the performance of their curriculum leadership. On the other hand, restricted by the conditions of rural economic development, rural teachers lack professional support for curriculum leadership development. Rural teachers receive far fewer training opportunities, material allocation, and curriculum resources than urban teachers. Under these circumstances, it is inevitable to expose shortcomings and deficiencies in the promotion of curriculum leadership awareness, the use of modern information technology, and the development and integration of curriculum resources.

### Rural Teachers Were Not Willing to Lead the Curriculum and Lacked Original Driving Force

Teachers’ leadership willingness, self-efficacy, and interpersonal skills all played positive roles in promoting rural teachers’ curriculum leadership. By further comparing standardized regression coefficient β, this study found that teachers’ leadership willingness was the most prominent factor affecting rural teachers’ curriculum leadership. If teachers showed strong leadership willingness, they could then take the initiative to lead the curriculum, otherwise, they would indulge in the status quo. What needs to be explained is that this kind of leadership willingness does not refer to the teachers’ administrative leadership, but their willingness to participate in curriculum leadership. Studies have confirmed that teachers’ motivation and willingness to be leaders partly decided whether they could successfully obtain leading roles (Meirink et al., 2020). Teachers hoped to participate more in curriculum leadership rather than other aspects of leadership, but due to administrative management and other reasons, many teachers were unwilling to participate in curriculum and teaching decision-making (Duke et al., 1980; Ho, 2010). The possible explanation is that willingness is the prerequisite for curriculum leadership. For rural teachers, although they teach and educate people in the countryside, they lack a sense of identity and local feelings and regard themselves as bystanders and outsiders. Most of them are young teachers with little work experience. Such young teachers are usually born in as well as growing up in cities so that they have little contact with rural members. This “natural” geographical difference prevents them from developing their enthusiasm for rural affairs and education life (Autti and Baeck, 2019). Fundamentally, they lack the essential awareness of taking root in the countryside, serving and dedicating to the countryside with their strength. Another possible explanation is that it is difficult for rural teachers to gain recognition from schools because they cannot implement curriculum leadership, which potentially affects their confidence in implementing curriculum leadership. Recognition within schools is critical to their leadership motivation and commitment to professional learning as well as classroom practice (Mukeredzi, 2016). If teachers feel undervalued, it may lead to frustration and less participation (Anderson, 2002). But for rural teachers, especially those with a relatively long teaching experience, are slow in accepting “new things” and have the low ability. In the long run, in the conflict between their efforts to gain group recognition and repeated frustration, their enthusiasm and self-confidence are constantly suppressed, and their sense of self-efficacy gradually decreases, which indirectly reduces their willingness to lead.

### Rural Teachers Were Limited in Their Self-Development Ability and Needed to Rely on the Power of the School Development Community

The results showed that there was a significant positive correlation between school common vision, teacher community as well as principal’s empowerment of teachers and rural teachers’ curriculum leadership. The shared vision of the school and the community of teachers were manifested as kinds of potential emotional supports, which played an important motivational effect on improving the curriculum leadership of rural teachers. Among them, according to the empirical analysis of influencing factors of rural teachers’ curriculum leadership, common vision within the school had a highly significant positive impact on the overall level of rural teachers’ curriculum leadership and its various sub-dimensions, which was the most explanatory factor in the school field and also an important factor for improving school effectiveness. This can be explained that schools with a good common goal can concentrate the power of all parties, encourage all members to coordinate with each other in daily education and curriculum leadership and enhance the awareness of common development. In other words, when school members, including principals, teachers, and other staff members can form a common vision, they will work closely under such an atmosphere to discuss the appropriate development of the school (Hart, 1994). Zhan et al. (2020) also pointed out in their study that if they developed the vision jointly, they would also tend to communicate the vision in a shared way (p.15), thus forming a stronger synergy to promote curriculum and school development. Conversely, schools lacking a unified vision will inhibit the generation of teacher curriculum leadership (Brooks et al., 2004), which will eventually make teachers lose the motivation to continue to grow and become stagnant due to the lack of clear goal guidance.

The teacher community was a supportive factor that affected the improvement of rural teachers’ curriculum leadership. The teacher community is the product of cooperation and communication among teachers, which is based on the consistent development vision and school culture characterized by democracy, cooperation, and trust. Interconnections between principals and teachers as well as interactions between teachers and colleagues were key factors in the successful development of leadership roles (Silva et al., 2000; Szeto and Cheng, 2018). The teacher community can effectively promote the development of rural teachers’ professional level, especially curriculum leadership. Through the community, they are no longer in a state of self-isolation but can promote the exchange and complementarity of high-quality resources, ideas, and concepts owned by each other. This explanation is confirmed in the study of Tonna and Bugeja (2018), within which educators expressed the eagerness for cooperative learning, and through cooperative learning, it was found that “teaching and learning can be enhanced when teachers collectively examine ineffective teaching practices, study new conceptions of teaching and learning, and support one another’s professional growth.” The results of this study once again confirmed the important role of the school’s common vision and the teacher community in improving the curriculum leadership of rural teachers. At the same time, interpersonal skills are an indispensable quality for teachers to conduct curriculum leadership and are the facilitating factor for the improvement of teachers’ curriculum leadership (O’Gorman and Hard, 2013). The more teachers communicate with colleagues, parents, and students, the more conducive to the implementation of curriculum leadership.

In addition, principal empowerment was a necessary condition for the generation of curriculum leadership of rural teachers, and it had a significant positive role in promoting it. According to the research results, if the principal could give teachers full trust, support encouragement, the autonomy of curriculum leadership and the opportunity to participate in curriculum decision-making, the confidence and determination of teachers in implementing curriculum leadership would be greatly enhanced. However, if the principal implemented vertical management and promoted teacher curriculum leadership by administrative orders, it might hinder the participation of rural teachers in curriculum leadership. Research by Wan and Leung (2021) proved this result, “whilst teacher autonomy and empowerment are marginalized, where ‘professional judgment’ in curriculum decision-making is not realized by future teachers who simply rely on the orders as requested” (p.19). Teachers have the most direct connection with the curriculum. Curriculum development should naturally be a bottom-up process, and teachers play a core role in curriculum development and leadership (Handler, 2010). Therefore, it is necessary to provide an external guarantee for the improvement of curriculum leadership of rural teachers to ensure that they exercise certain curriculum leadership.

## Conclusion and Implications

The curriculum leadership of rural teachers is a key force to promote the development of rural education and promote rural revitalization. Therefore, it is necessary to strengthen the leadership of rural teachers (Rodrigues et al., 2005). Through a large-scale empirical investigation, this study found that the overall level of curriculum leadership of rural teachers in China was not high, which could be attributed to the comprehensive effect of teachers’ individual field factors and school field factors. To solve this problem, we need to start with the realistic factors that promote or hinder the development of curriculum leadership of rural teachers and break through their weak points gradually. Given that the development of rural education and the improvement of the professional level of rural teachers are widely accepted by all countries in the world, and Chinese rural teachers occupy a considerable size and position in the global rural teachers’ team, exploring strategies for improving the curriculum leadership of Chinese rural teachers will also provide some enlightenment on relevant policies and practices of other countries. Therefore, based on empirical research conclusions, this paper puts forward the following suggestions:

First of all, the willingness of rural teachers to lead the curriculum should be cultivated and their awareness of self-identity should be enhanced. As important members of the rural organizational structure, rural teachers need to fully recognize their leadership and its possible influence, and at the same time understand their role in educational reform (Jackson et al., 2010). The fundamental approach lies in the solution of teachers’ inability or unwillingness to participate in curriculum leadership. In this regard, it is necessary to cultivate the willingness of curriculum leadership as the starting point to enhance rural teachers’ awareness of curriculum leadership and their identity and make it clear that curriculum leadership is a test of their comprehensive capabilities such as value guidance, teamwork, curriculum planning, and cultural construction. Therefore, teachers should realize the transition from the traditional curriculum implementer role to curriculum developer in curriculum leadership, and assume more responsibility in the curriculum decision-making of students’ learning (Marsh, 1997; Harris, 2003), thereby further helping them eliminate resisting emotions.

Secondly, a common vision for school development should be built and rural teachers’ mission of realizing rural revitalization should be strengthened. The common vision plays an important role in guiding people’s actions. To achieve the goal of rural education development, rural schools and rural teachers should be linked with the development of the entire village, cast a shared vision for the school based on rural revitalization, integrate into rural life, and take concerted actions to realize the educational mission of promoting rural revitalization and grow from “teaching in the countryside” to “teaching for the countryside.”

Thirdly, a diversified participatory curriculum leadership community should be established, and paired assistance for rural teachers’ curriculum leadership should be carried out. For rural teachers, the most effective way to change the status quo of weak curriculum leadership is to promote the transformation of teachers’ teaching from individual “I” to group “we,” and build a multi-participatory community of curriculum leadership of rural teachers. The leadership community contains not only the cooperative groups formed by various teachers in the school, but also groups formed by the paired teaching of experienced teachers and novice teachers, counterpart assistance between high-quality urban schools and weak rural schools, and the communication between rural schools, teachers and local educational organizations, to provide various rich and high-quality resources for rural teachers’ curriculum implementation and help rural teachers to become innovative and professional enablers in serving rural revitalization. It is worth noting that in the process of building a curriculum leadership community, we should pay full attention to the practical differences between rural and urban teachers. Based on integrating high-quality resources, we should improve the “place-based” curriculum leadership of rural teachers. That is, to promote rural teachers to enhance their understanding of local culture, and to carry out local curriculum leadership in combination with regional characteristic resources.

Finally, rural teachers should be empowered to implement curriculum leadership and provided with exogenous support. As revealed in this study, principal empowerment is an important condition for rural teachers to exert and develop their curriculum leadership. “Curriculum decision-making, therefore, is not the sole responsibility of a few key personnel appointed by the school authority but a process (or a phenomenon) to be shared equally among all teachers in the school” (Law et al., 2007, p.145). In this regard, the power of the implementation of curriculum leadership for rural teachers must be loosened to ensure that rural teachers get sufficient activity space and discourse power in the process of curriculum planning, implementation, and evaluation, allowing teachers to have the right and autonomy to select and develop curricula (Shen et al., 2020), so that rural teachers can lead their colleagues to reconstruct the local curriculum system, make use of local school-based curriculum materials to create a lively local classroom.

There are still some limitations in this study, which also outline the direction of future research. Firstly, this study presents the current situation of rural teachers’ curriculum leadership from the perspective of urban-rural comparison, which may exacerbate the stereotype of rural teachers. Follow-up research will combine with qualitative data and adopt a more positive and in-depth perspective to reveal the uniqueness and “place-based” wisdom of rural teachers’ curriculum leadership. Secondly, although the measurement instruments of this study have undergone expert consultation, trial investigation, and multiple rounds of revision, it is difficult to completely avoid the problem of heterogeneity due to the diversity of the participants. Future research will further improve the research instruments based on paying more attention to urban-rural equity and regional equity. Thirdly, this study is intended to broadly explore the influencing factors of rural teachers’ curriculum leadership, which may limit in-depth attention to specific important factors. Later on, we will focus on the more important factors revealed by this paper, such as teachers’ willingness to lead the curriculum, teachers’ interpersonal skills, and the school common vision, and use a mediation/moderation model to explore the specific mechanisms of their effects. At the same time, since the selection of teachers’ individual field factors in this study mainly considers more internal attributes such as teachers’ quality and ability, some important external attributes are excluded, such as teachers’ professional titles and positions. These factors were used as control variables in this study. It is undeniable that these factors also have an impact on the curriculum leadership of rural teachers. Future research will also consider these factors as independent variables. In addition, an international comparative study of rural teachers’ curriculum leadership will be carried out in future research to fully demonstrate the commonality and particularity of rural teachers’ curriculum leadership in China and different countries.

## Data Availability Statement

The raw data supporting the conclusions of this article will be made available by the authors, without undue reservation.

## Ethics Statement

Ethical review and approval was not required for the study on human participants in accordance with the local legislation and institutional requirements. The patients/participants provided their written informed consent to participate in this study.

## Author Contributions

XW, JC, and WY contributed to the ideas of educational research. FX contributed to the contacting participant and collection of data. XW, JC, WY, YZ, and FX contributed to the data analysis, design of research methods, and tables. XW, JC, and YZ participated in writing and revision. All authors have read and agreed to the published version of the manuscript.

## Conflict of Interest

The authors declare that the research was conducted in the absence of any commercial or financial relationships that could be construed as a potential conflict of interest.

## Publisher’s Note

All claims expressed in this article are solely those of the authors and do not necessarily represent those of their affiliated organizations, or those of the publisher, the editors and the reviewers. Any product that may be evaluated in this article, or claim that may be made by its manufacturer, is not guaranteed or endorsed by the publisher.
