# Supplementary material for: Curriculum Leadership of Rural Teachers: Status Quo, Influencing Factors and Improvement Mechanism-Based on a Large-Scale Survey of Rural Teachers in China
Source: Front Psychol. 2022 Mar 11;13:813782. doi: 10.3389/fpsyg.2022.813782 (PMC8962949; doi:10.3389/fpsyg.2022.813782)
Supplement: Supplementary file 1 [file Data_Sheet_1.docx]

**Appendix**

**Appendix A**

**Questionnaire on Curriculum Leadership of Chinese Primary and Secondary School Teachers**

1. Your gender is:

(1) Male (2) Female

2. When did you start working as a teacher?

3. Your school is located at:

(1) Ethnic regions (2) Non-Ethnic regions

4. Your school is located at:

(1) Urban area (2) Rural area

5. The nature of your school is:

(1) Public school (2) Private school

6. Compared with other schools in this region (city/town/country), what is the ranking of your school in recent two years?

(1) Medium and below medium (2) Above medium (3) The best

7. What subject (s) are you teaching now? (You can choose more than one answer)

(1) Chinese (2) Math (3) English (4) Physics (5) Chemistry (6) Biology

(7) Ethics and Rule of Law / Ideological and Political Course (8) Geography

(9) History/ History and Society (10) Music (11) Art (12) Sports & Health

(13) Information Technology (14) Science (15) Others (please note here )

8. What is your current teaching period?

(1) Primary school (2) Middle school (3) High school

9. Your highest degree is:

(1) Junior high school and below (2) Technical secondary school (3) Technical school

(4) Vocational High School (5) Ordinary high school (6) College degree

(7) Bachelor degree (8) Master degree (9) Doctoral degree

10. Do you have a professional background of teacher education?

(1) Yes (2) No

11. Your current title is:

(1) Unrated (2) Third-level teacher (3) Secondary-level teacher

(4) First-level teacher (5) Advanced teacher (6) Senior teacher

12. Your current post (s) is/are: (You can choose more than one answer)

(1) None (2) Class teacher (3) Lesson preparation team leader

(4) Teaching-research team leader (5) Grade manager (6) Dean/Deputy Dean

(7) Vice-principal (8) Principal (9) Others (please note here )

13. What is the highest award you have received for teaching?

(1) Not yet awarded (2) School level awards (3) County (district) level awards

(4) Municipal awards (5) Provincial awards (6) National awards

14. Please mark “√” on the column that best meets your actual situation.

| **Items** | **Strongly Disagree** | **Disagree** | **Average** | **Agree** | **Strongly Agree** |
| --- | --- | --- | --- | --- | --- |
| (1) You have mastered the advanced and frontier educational concepts and curriculum ideas. |  |  |  |  |  |
| (2) You have mastered the latest national and local curriculum policies, curriculum plans, and subject curriculum standards. |  |  |  |  |  |
| (3) You can communicate curriculum policies to students and parents in a timely and appropriate manner. |  |  |  |  |  |
| (4) You lead students, parents, or colleagues to work together to form and implement a curriculum vision based on key competencies. |  |  |  |  |  |
| (5) You always carry out a unit instructional design based on your overall understanding of the key competencies and the students in your class. |  |  |  |  |  |
| (6) You make full use of existing curriculum resources (schools, communities, Internet, etc.) to maximize the effect of cultivating students’ key competencies. |  |  |  |  |  |
| (7) You discuss and share curriculum schemes based on key competencies with your colleagues. |  |  |  |  |  |
| (8) In your class, most students are highly concentrated and speak enthusiastically. |  |  |  |  |  |
| (9) You pay attention and respond to the learning needs of different students. |  |  |  |  |  |
| (10) You make good use of modern information technology in the implementation of the curriculum to guide students to gradually achieve self-development. |  |  |  |  |  |
| (11) You make good use of the products of teaching research in the implementation of the curriculum to guide students to gradually realize independent development. |  |  |  |  |  |
| (12) You often adjust the teaching progress or method in time according to the teaching reality. |  |  |  |  |  |
| (13) You evaluate the quality of curriculum practice and student development based on the effectiveness of the curriculum vision. |  |  |  |  |  |
| (14) You conduct curriculum evaluation based on teaching data, growth records, and other evidence. |  |  |  |  |  |
| (15**)** You carry out information collection, teaching diagnosis, or curriculum evaluation simultaneously in the teaching process. |  |  |  |  |  |
| (16) You will optimize the curriculum planning based on the results of curriculum reflection. |  |  |  |  |  |
| (17) You meet the individual development needs of students by developing a school-based curriculum (undeveloped curriculum please select “strongly disagree”). |  |  |  |  |  |
| (18) You are actively developing new curriculum resources to enhance the cultivation effect of students’ key competencies (undeveloped curriculum please select “strongly disagree”). |  |  |  |  |  |
| (19) You develop curriculum scientifically according to discipline curriculum standards and other relevant regulations (undeveloped curriculum please select “strongly disagree”). |  |  |  |  |  |
| (20) After developing a new curriculum, you always invite experts to check the quality (undeveloped curriculum please select “strongly disagree”). |  |  |  |  |  |
| (21) You understand the key competencies of Chinese student development. |  |  |  |  |  |
| (22) You understand the key competencies of the teaching subject. |  |  |  |  |  |
| (23) You have a certain understanding of teacher curriculum leadership. |  |  |  |  |  |
| (24) You know how to use curriculum leadership to cultivate students’ key competencies. |  |  |  |  |  |
| (25) You know the significance of teacher curriculum leadership for developing students’ key competencies. |  |  |  |  |  |
| (26) You believe that teachers as a professional group can and should participate in curriculum leading, management, and decision-making. |  |  |  |  |  |
| (27) You believe that only with administrative positions can teachers enact curriculum leading, management, and decision making. |  |  |  |  |  |
| (28) You believe that teachers who actively participate in the construction of the curriculum community are “curriculum leaders”, even if they do not own administrative positions. |  |  |  |  |  |
| (29) You believe you can and should be involved in curriculum leading, management, and decision-making. |  |  |  |  |  |
| (30) You have a strong will to lead the curriculum. |  |  |  |  |  |
| (31) You fully value the professional advice of your colleagues in making curriculum decisions. |  |  |  |  |  |
| (32) You believe that with the necessary effort you can effectively accomplish all your tasks. |  |  |  |  |  |
| (33) Even if you are busy at work, you can still plan your daily work reasonably. |  |  |  |  |  |
| (34) You always get support from and cooperation with students, parents, colleagues, leaders, or experts in your curriculum practice. |  |  |  |  |  |
| (35) Your school has a high degree of compatibility between the school philosophy, training goals (educational goals), and curriculum aims. |  |  |  |  |  |
| (36) Your school has formed a cultural atmosphere of mutual trust. |  |  |  |  |  |
| (37) In your school, teachers help each other and develop cooperatively. |  |  |  |  |  |
| (38) In your school, the management level will take teachers’ opinions into account when making curriculum decisions. |  |  |  |  |  |
| (39) Your principal provides sufficient conditions for the professional growth of teachers. |  |  |  |  |  |
| (40) Your principal often exchanges ideas on curriculum improvement with different teachers. |  |  |  |  |  |
| (41) Your principal provides opportunities for teachers who do not have positions but have outstanding professional abilities to participate in curriculum management and decision-making. |  |  |  |  |  |
| (42) Your principal will give appropriate rewards to teachers for their efforts. |  |  |  |  |  |
